# Evaluation of myocardial strain using cardiovascular magnetic resonance imaging in patients with β-thalassemia major

**DOI:** 10.1186/s44348-024-00033-2

**Published:** 2024-09-02

**Authors:** Sung Min Ko

**Affiliations:** Department of Radiology, Wonju Severance Christian Hospital, Yonsei University Wonju College of Medicine, Wonju, Republic of Korea

β-Thalassemia major, also known as Cooley anemia, is a severe hereditary blood disorder characterized by impaired synthesis of the beta-globin subunit of hemoglobin. This defect leads to abnormal hemoglobin production, causing chronic hemolytic anemia. β-Thalassemia major patients need regular blood transfusion during their whole life. Frequent blood transfusions lead to inevitable myocardial iron overload (MIO), which can lead to cardiomyopathy, arrhythmias, and heart failure, representing a major cause of morbidity and mortality [1]. Cardiovascular magnetic resonance imaging (CMR) is a useful imaging tool to evaluate MIO. T2* CMR is considered the gold standard for noninvasive detection and quantification of MIO. This allows early detection of MIO in patients with β-thalassemia before clinical signs of cardiac dysfunction appear [2, 3]. However, even patients with similar T2* values ​​may present with varying degrees of cardiomyopathy. The pathophysiological complexity of iron-induced cardiac injury in β-thalassemia major necessitates the use of advanced imaging techniques for the early detection of myocardial dysfunction [4–7].

Myocardial strain refers to the change in length of myocardial fibers between their relaxed and contractile states. Describing left ventricular (LV) strain in three dimensions using normal and shear strain provides a comprehensive assessment of myocardial mechanics. The assessment of LV strain components (longitudinal, circumferential, and radial) offers a detailed evaluation of myocardial function. Longitudinal strain serves as an early marker of dysfunction, circumferential strain provides insights into the transmural and epicardial involvement, and radial strain indicates advanced disease stages. Measuring myocardial strain allows for the detection of subtle myocardial dysfunction that may not be apparent with commonly used LV ejection fraction [8, 9]. While speckle tracking echocardiography is commonly used for myocardial strain, it may be limited by acoustic windows and image quality [8, 9]. CMR feature tracking (FT) is an advanced imaging technique used to evaluate myocardial strain by utilizing postprocessing of cine MR images routinely acquired during CMR scans. Therefore, CMR-FT is becoming increasingly available and is now frequently combined with LV volume assessment, parametric mapping, gadolinium delayed enhancement, and myocardial perfusion in a single examination, providing a comprehensive assessment of myocardial function and tissue characteristics [10–12]. Correlation between strain values ​​obtained from speckle tracking echocardiography and CMR-FT may vary due to technical differences, postprocessing techniques, resolution constraints, and patient-specific factors [13, 14]. Iron deposition in the subepicardial layer in patients with β-thalassemia major primarily affects the circumferential strain. Longitudinal strain may be affected as iron deposition progresses, and radial strain is usually affected later, when iron overload causes further progression and transmural involvement of the myocardial wall [15–17]. Several studies have investigated the correlation between CMR-FT strain values and T2* CMR values, specifically focusing on their roles in assessing contractile abnormalities in patients with β-thalassemia major [18, 19].

In this issue of the *Journal of Cardiovascular Imaging*, Batouty et al. [20] assessed the ability of CMR-FT in the early detection of LV systolic dysfunction in β-thalassemia major patients and correlated it with the degree of MIO measured by CMR T2* values. The study cohort consisted of 57 patients with β-thalassemia major and 20 healthy controls. MIO was diagnosed by CMR T2* values ​​based on LV septum less than 20 ms. CMR was performed using a 1.5-T magnet, and two-dimensional FT analysis was performed offline using dedicated CMR software. This study had several results. First, LV strain parameters (global circumferential stain [GCS] and global radial strain [GRS]) were significantly lower in thalassemia patients compared to the control group. Second, thalassemia patients without MIO showed significantly lower GCS and GRS compared to the control group. Third, there was significant correlation between GCS and GRS and T2* values even in absence of significant correlation with LV ejection fraction. Fourth, GCS and GRS values were lower in the basal and mid myocardial segments compared to the apical segments. Interestingly there was no significant difference between patients and the control group regarding global longitudinal strain (GLS) values, and there was no significant correlation between GLS values and T2* values. There were no significant differences between patients with and without MIO in GLS, GCS, and GRS values. These results differ from other previous studies and suggest the pathophysiological complexity of iron-induced cardiac injury and the influence of coexisting factors on the myocardium [19, 21, 22]. Additional studies are needed to explain these findings in larger patient populations and explore the mechanisms underlying the various relationships.

However, these interesting results of the current study need to be interpreted in the context of several limitations, including the small number of β-thalassemia major patients (57, especially with 11 MIO patients), no follow-up CMR studies, single-vendor MR application, and single-center study. Overall, this study highlights the important role of CMR-FT strain parameters as markers for early detection of LV systolic dysfunction in patients with β-thalassemia major, especially in combination with CMR T2* values.

## Data Availability

Not applicable.

## Abbreviations

CMR: Cardiovascular magnetic resonance
FT: Feature tracking
GCS: Global circumferential stain
GLS: Global longitudinal strain
GRS: Global radial strain
LV: Left ventricular
MIO: Myocardial iron overload
MR: Magnetic resonance

## Data Availability

No datasets were generated or analysed during the current study.
